# Comparative Diagnostic Accuracy of Spheno-Occipital Synchondrosis and Cervical Vertebral Maturation Index in Predicting Mandibular Growth Parameters in Adolescents: A Systematic Review and Meta-Analysis

**DOI:** 10.7759/cureus.108572

**Published:** 2026-05-09

**Authors:** Akshata Awachat, Ananya Hazare

**Affiliations:** 1 Orthodontics and Dentofacial Orthopaedics, Ranjeet Deshmukh Dental College and Research Centre, Nagpur, Nagpur, IND

**Keywords:** cervical vertebral maturation index, cone-beam computed tomography, mandibular growth, skeletal maturity, spheno-occipital synchondrosis, systematic review and meta-analysis

## Abstract

This systematic review and meta-analysis evaluated and compared the diagnostic utility of spheno-occipital synchondrosis maturation and the cervical vertebral maturation index for identifying mandibular growth parameters in adolescents. Longitudinal and mixed-longitudinal human studies that assessed spheno-occipital synchondrosis maturation and/or the cervical vertebral maturation index as index tests against mandibular growth parameters as the reference standard were considered eligible. Cross-sectional studies without a longitudinal growth reference and studies lacking extractable diagnostic accuracy data were excluded. A comprehensive search of PubMed/MEDLINE, Embase, Scopus, Web of Science, and the Cochrane Library was performed from database inception to the final search date, and grey literature was additionally screened through ProQuest Dissertations & Theses, selected university repositories, ClinicalTrials.gov, and the World Health Organization International Clinical Trials Registry Platform. Risk of bias and applicability concerns were independently assessed using Quality Assessment of Diagnostic Accuracy Studies-2 (QUADAS-2). Sensitivity estimates were derived from 2 × 2 contingency tables and pooled using a random-effects model, while the association between spheno-occipital synchondrosis maturation and cervical vertebral maturation index stages was synthesized through correlation meta-analysis. Six studies involving adolescents approximately 10 to 19 years of age met the inclusion criteria. Three longitudinal studies were included in the diagnostic accuracy meta-analysis for the cervical vertebral maturation index, and three cross-sectional studies contributed four datasets to the correlation meta-analysis examining the relationship between spheno-occipital synchondrosis maturation and the cervical vertebral maturation index. The cervical vertebral maturation index demonstrated moderate diagnostic performance for identifying mandibular growth phases, although sensitivity estimates varied notably across studies. Spheno-occipital synchondrosis maturation showed a strong pooled association with mandibular growth and skeletal maturation, with a random-effects correlation of approximately 0.8, although heterogeneity was considerable. Overall, both measures appear clinically informative for estimating mandibular growth timing in adolescents, with spheno-occipital synchondrosis maturation showing a strong relationship with skeletal maturation and the cervical vertebral maturation index demonstrating moderate diagnostic value for identifying phases of mandibular growth. No external funding was received. This review was registered in PROSPERO (CRD420251175309).

## Introduction and background

Rationale

Accurate assessment of skeletal maturity is fundamental in orthodontics, particularly for optimizing the timing of growth-modification therapies during adolescence. Mandibular growth exhibits considerable inter-individual variability, and inappropriate timing of treatment may compromise growth modulation outcomes [1-3]. Several biological indicators have been proposed to estimate skeletal maturity, among which the cervical vertebral maturation index (CVMI) is the most widely used due to its availability on routine lateral cephalograms [4-6]. The CVMI was originally introduced by Lamparski and later refined by Hassel and Farman, and subsequently modified by Baccetti, Franchi, and McNamara to improve its clinical applicability in identifying the pubertal growth peak [4-6]. However, concerns remain regarding its diagnostic accuracy, reproducibility, and ability to predict individual mandibular growth phases [7-9].

In recent years, spheno-occipital synchondrosis (SOS) maturation assessed using cone-beam computed tomography (CBCT) has emerged as a potential alternative indicator of skeletal growth. SOS fusion is a biologically regulated process closely associated with cranial base development and pubertal maturation [10-12]. Preliminary studies have demonstrated a strong association between SOS fusion stages and skeletal maturation indicators, including mandibular growth patterns [13-15]. However, the diagnostic performance of SOS relative to established maturity indicators has not been systematically synthesized.

Despite increasing interest in SOS-based assessment, existing studies are heterogeneous in design and methodology, and no prior systematic review has comprehensively evaluated the diagnostic accuracy of SOS or CVMI using longitudinal mandibular growth as the reference standard. Therefore, a systematic review and meta-analysis of diagnostic test accuracy is warranted to clarify the clinical utility of these indices [16].

Clinical role of the index tests

The intended clinical role of both index tests - CVMI and SOS maturation - is to serve as noninvasive diagnostic tools for identifying mandibular growth phases in growing subjects. CVMI is routinely used to guide the timing of orthopedic interventions and growth modification; however, it is derived from two-dimensional imaging and may be influenced by observer subjectivity and morphological variability [4,7,8].

In contrast, SOS maturation is assessed using three-dimensional CBCT imaging, allowing direct visualization of the cranial base and potentially improving staging accuracy [11,13]. The biological rationale for SOS as an index test lies in its close developmental relationship with cranial base growth and facial skeletal maturation [10,11]. A clinically acceptable diagnostic test should reliably differentiate active mandibular growth from non-growth phases with sufficient sensitivity and specificity to inform treatment timing decisions [17,18].

This systematic review addressed the following clinical question: in adolescent patients undergoing growth assessment (Population), how accurately do the CVMI and SOS maturation (Index tests) identify the timing of mandibular growth when compared with longitudinal mandibular growth assessment or established skeletal maturation standards (Reference standards), and what is the strength of their association with mandibular growth parameters (Outcomes)?

Objectives

This systematic review and meta-analysis aimed to evaluate the diagnostic accuracy and associative performance of SOS maturation and the CVMI for assessing mandibular growth timing in adolescents. The review specifically examined the diagnostic performance of the CVMI for identifying the mandibular growth peak against longitudinal mandibular growth measurements as the reference standard and assessed the association between SOS maturation stages and CVMI stages through correlation meta-analysis when direct diagnostic accuracy data were unavailable.

## Review

Methods

Protocol and Registration

This systematic review and meta-analysis were conducted in accordance with the Preferred Reporting Items for Systematic Reviews and Meta-Analyses for Diagnostic Test Accuracy (PRISMA-DTA) guidelines [19]. The review protocol was prospectively registered in the International Prospective Register of Systematic Reviews PROSPERO (registration number - CRD420251175309). The protocol defined the research question, eligibility criteria, outcomes, and planned methods of analysis and was followed without deviation.

Eligibility Criteria

Studies were considered eligible if they met the following criteria.

Participants: Human subjects in the mixed or permanent dentition stages with available skeletal maturity or growth assessment data.

Index tests: CVMI assessed on lateral cephalograms and/or SOS maturation assessed using CBCT.

Reference standard: Longitudinal mandibular growth assessment based on serial cephalometric measurements, such as annual increments in mandibular length.

Target condition: Presence or absence of active mandibular growth, including the mandibular growth peak.

Study design: Longitudinal studies were included for diagnostic test accuracy analysis, whereas cross-sectional or mixed longitudinal studies reporting associations between SOS and CVMI were included for correlation meta-analysis.

Report characteristics: Peer-reviewed articles published in English with no restrictions on publication year.

Studies were excluded if they lacked a longitudinal growth reference standard, relied solely on cross-sectional data for diagnostic accuracy assessment, involved non-human subjects, or did not provide sufficient information to construct 2 × 2 contingency tables.

Information Sources

A comprehensive electronic search was conducted in the following databases: PubMed/MEDLINE, Embase, Scopus, Web of Science, and the Cochrane Library from inception to 30 June 2025. Additional searches were performed in Google Scholar (first 200 results only) and trial registries including ClinicalTrials.gov and the WHO International Clinical Trials Registry Platform (ICTRP). Grey literature sources included ProQuest Dissertations & Theses and selected university repositories. Reference lists of included studies and relevant reviews were hand-searched to identify additional eligible articles.

Search Strategy

Search strategies combined controlled vocabulary and free-text terms related to skeletal maturation, mandibular growth, cervical vertebral maturation, and SOS. Boolean operators (“AND”, “OR”) were used to combine terms appropriately. A representative search strategy was developed for PubMed/MEDLINE and adapted for other databases. The PubMed search included combinations of the following terms: (“cervical vertebral maturation” OR CVMI) AND (“spheno-occipital synchondrosis” OR SOS) AND (“mandibular growth” OR “growth peak” OR “skeletal maturation”). The full electronic search strategies for all databases are provided in the Supplementary Material to ensure reproducibility.

Study Selection

**Figure 1:**
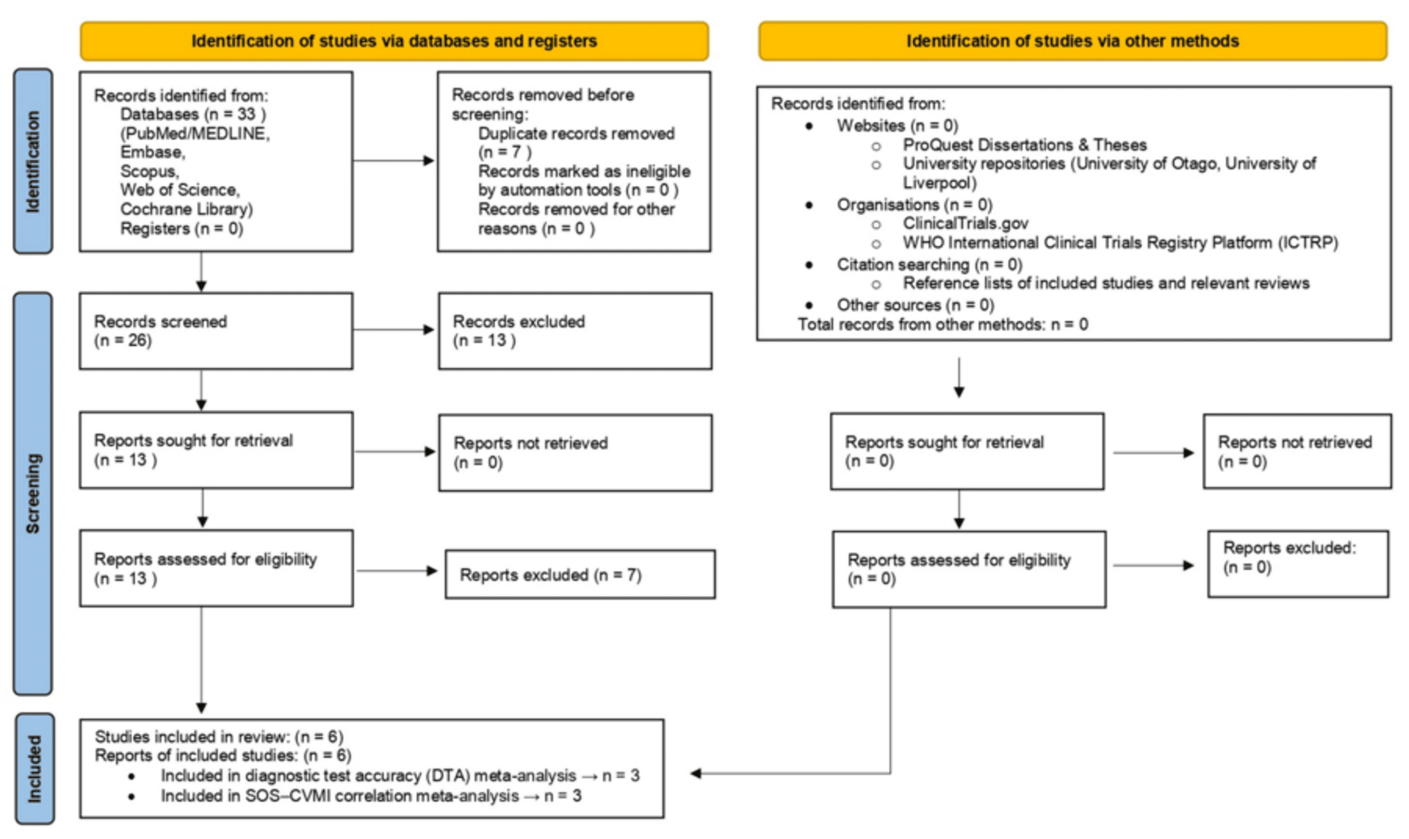
Preferred Reporting Items for Systematic Reviews and Meta-Analyses (PRISMA) 2020 flow diagram illustrating the identification, screening, eligibility assessment, and inclusion of studies in the systematic review and meta-analyses. Seven full-text articles were excluded after eligibility assessment. Six studies met the inclusion criteria, of which three were included in the diagnostic test accuracy meta-analysis and three in the correlation meta-analysis. In the correlation meta-analysis, four datasets were analyzed because Kocasarac et al. contributed separate male and female subgroup data [16].

All identified records were imported into reference management software, and duplicate records were removed. Two reviewers independently screened titles and abstracts for eligibility. Full texts of potentially relevant studies were retrieved and assessed independently. Disagreements at any stage were resolved by discussion, and when necessary, consultation with a third reviewer. The study selection process is summarized using a PRISMA 2020 flow diagram (Figure 1). Full-text articles were then assessed independently for eligibility. Any disagreements at either stage were resolved through discussion and consensus.

Data Collection

Data extraction was performed independently by two reviewers using a standardized and piloted data extraction form. Extracted information included study characteristics, participant demographics, index test details, reference standards, and diagnostic accuracy data. When required data were missing or unclear, attempts were made to contact study authors for clarification. Extracted data were cross-checked for accuracy, and disagreements were resolved by consensus.

Definitions for Data Extraction

The target condition was defined as the presence of active mandibular growth or mandibular growth peak as determined by longitudinal growth measurements. Index test positivity thresholds were defined according to the original study criteria for CVMI stages or SOS fusion stages. Diagnostic accuracy data were extracted in the form of true positives, false positives, false negatives, and true negatives at the patient level.

Risk of Bias and Applicability

The methodological quality of the included studies and concerns regarding applicability were independently assessed by two reviewers using the Quality Assessment of Diagnostic Accuracy Studies-2 (QUADAS-2) tool [20]. This instrument evaluates risk of bias across four domains: patient selection, index test, reference standard, and flow and timing, as well as concerns regarding applicability in the first three domains. Each domain was rated as “low,” “high,” or “unclear” risk of bias in accordance with published guidance. Disagreements between reviewers were resolved by consensus discussion.

Diagnostic Accuracy Measures

The primary diagnostic accuracy measures were sensitivity and specificity for identifying mandibular growth. Diagnostic accuracy was assessed on a per-patient basis. Secondary outcomes included pooled correlation coefficients between SOS fusion stages and CVMI stages.

Synthesis of Results

For diagnostic test accuracy, sensitivity and specificity were calculated for each study from extracted 2×2 data. Due to the limited number of eligible longitudinal studies and inconsistent reporting of specificity thresholds, pooled diagnostic accuracy analyses focused on sensitivity estimates rather than full bivariate hierarchical summary receiver operating characteristic (HSROC) modeling. Pooled sensitivity estimates were obtained using random-effects meta-analysis models. Between-study heterogeneity was assessed using the I² statistic.

For correlation outcomes, correlation coefficients were transformed using Fisher’s z transformation and pooled using random-effects models. Analyses were conducted separately for diagnostic accuracy and correlation outcomes to account for differences in study design and data structure.

Meta-Analysis Methods

Meta-analyses were performed using standard statistical software. Separate random-effects models were applied for sensitivity and correlation analyses. Forest plots were generated to visualize individual and pooled estimates. Statistical significance was set at P < 0.05.

Additional Analyses

Pre-specified subgroup analyses based on study design and index test type were planned; however, these analyses were limited by the small number of included studies. Sensitivity analyses were performed by excluding studies with high risk of bias to assess the robustness of pooled estimates.

Results

Study Selection

The electronic search identified 33 records from core databases (PubMed/MEDLINE, Embase, Scopus, Web of Science, and the Cochrane Library). After removal of seven duplicate records, 26 records were screened at the title and abstract level. Of these, 13 records were excluded for not meeting the eligibility criteria.

Thirteen full-text articles were retrieved and assessed for eligibility. Following full-text assessment, seven studies were excluded for reasons including absence of a longitudinal mandibular growth reference standard, cross-sectional study design only, and insufficient data to construct 2×2 diagnostic accuracy tables.

A total of six studies fulfilled the inclusion criteria and were included in the systematic review. Among these, three studies were eligible for diagnostic test accuracy (DTA) meta-analysis, and three studies were included in the SOS-CVMI correlation meta-analysis. For the correlation meta-analysis, four datasets were analyzed because sex-specific data from Kocasarac et al. [16] were treated as independent male and female subgroups.

Study Characteristics

The characteristics of the included studies are summarized in Table 1. The included studies comprised mixed longitudinal and longitudinal observational designs conducted in orthodontic or academic clinical settings. Study populations consisted of growing children and adolescents undergoing growth assessment.

**Table 1:** Characteristics of studies included in the systematic review. NR = not reported. CVMI = cervical vertebral maturation index; SOS = spheno-occipital synchondrosis; CBCT = cone-beam computed tomography; DTA = diagnostic test accuracy Only longitudinal studies with extractable 2×2 data were included in the diagnostic test accuracy meta-analysis. Kocasarac et al. contributed two non-overlapping sex-specific datasets (male and female) to the correlation meta-analysis but was counted as one included study in the systematic review

Study (Author, Year)	Country / setting	Study design	Population	Sample size (N)	Age (years)	Sex (M/F)	Index test	Imaging modality	Reference standard / comparator	Primary outcome	Included in synthesis
Perinetti et al., 2016 [9]	Orthodontic growth cohort	Longitudinal	Growing adolescents	102	NR	NR	CVMI	Lateral cephalogram	Longitudinal mandibular growth (serial cephalometrics)	Mandibular growth peak identification	DTA meta-analysis
Perinetti et al., 2018 [10]	Oregon & Burlington Growth Studies	Longitudinal	Growing subjects with annual records	250	~9-16	24/26	CVMI	Lateral cephalogram	Longitudinal mandibular growth (annual Co-Gn increments)	Mandibular growth peak identification	DTA meta-analysis
Morris et al., 2019 [11]	Orthodontic clinic sample	Longitudinal	Growing orthodontic patients	167	NR	NR	CVMI	Lateral cephalogram	Longitudinal mandibular growth	Mandibular growth peak identification	DTA meta-analysis
Fernández-Pérez et al., 2016 [15]	USA & Spain (university + private clinics)	Cross-sectional	Orthodontic patients	315	6-23	167/14 8	SOS fusion stage	CBCT	CVMI (Baccetti method)	SOS-CVMI association	Correlation meta-analysis
Fayad et al., 2020 [17]	Orthodontic clinic	Cross-sectional	Orthodontic patients	117	8-18	NR	SOS fusion stage	CBCT	CVMI (lateral cephalogram )	SOS-CVMI association	Correlation meta-analysis
Kocasarac et al., 2017 [16]	Turkey (radiology/orthodontic)	Cross-sectional	Adolescents & young adults	116	8-28	43/73	SOS fusion stage	CBCT	CVM (Hassel & Farman)	SOS-CVMI association (sex specific)	Correlation meta-analysis

For the diagnostic accuracy analysis, the index test was the CVMI, and the reference standard was longitudinal mandibular growth assessment, typically derived from serial cephalometric measurements. For the correlation analysis, the index test was SOS maturation stage, and the comparator was CVMI or mandibular growth indicators.

Sample sizes ranged from 43 to 315 participants, and publication years ranged from 2016 to 2020. Funding sources were either institutional or not explicitly reported.

In the study by Kocasarac et al., sex-specific correlation data were reported separately for male and female participants [16]; therefore, these subgroups were treated as independent datasets for the purposes of the correlation meta-analysis.

Risk of Bias and Applicability

**Figure 2:**
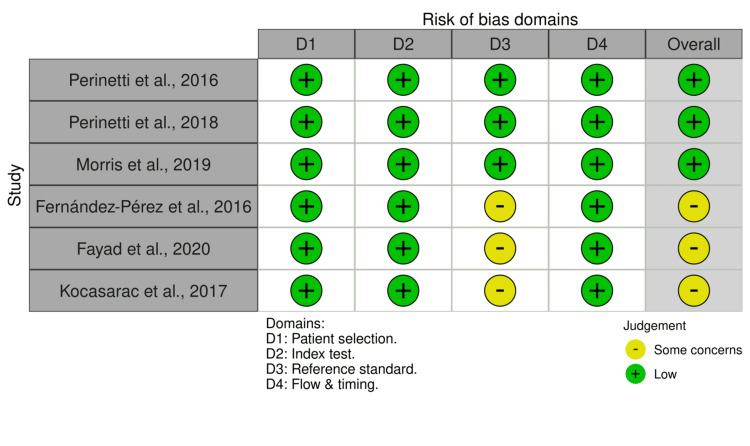
Risk of bias and applicability assessment of included studies using the Quality Assessment of Diagnostic Accuracy Studies-2 (QUADAS-2) tool. Green indicates low risk of bias or low concern regarding applicability; yellow indicates some concerns.

**Figure 3:**
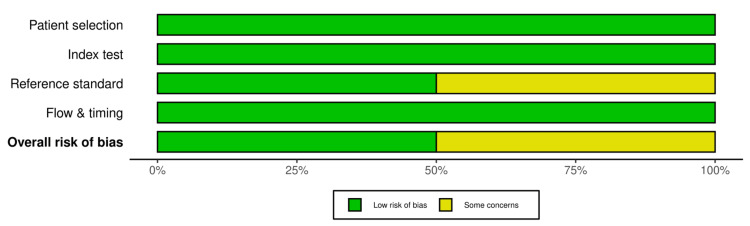
Summary of risk of bias across included studies assessed using the Quality Assessment of Diagnostic Accuracy Studies-2 (QUADAS-2) tool. Bars represent the proportion of studies judged as low risk of bias or having some concerns for each domain.

Risk of bias and applicability concerns were assessed using the QUADAS-2 tool [20]. Overall, the included studies demonstrated low risk of bias across most domains (Figures 2, 3). All studies were judged to have low risk of bias for patient selection, index test, and flow and timing.

Some concerns regarding the reference standard were identified in cross-sectional studies evaluating SOS fusion, where indirect comparators rather than longitudinal mandibular growth measurements were used. Applicability concerns were generally low across all domains, reflecting good alignment between the included studies and the clinical question of orthodontic growth assessment.

Results of Individual Studies

For each of the three studies included in the diagnostic test accuracy meta-analysis, 2×2 contingency tables were constructed based on reported or derived true positive, false positive, false negative, and true negative values (Table 2). Individual study sensitivity estimates ranged from 24.0% to 86.4%, demonstrating substantial between-study variability. Reported specificity estimates varied across studies, reflecting differences in study populations and CVMI threshold definitions.

**Table 2:** Diagnostic accuracy data (true positives, false positives, false negatives, and true negatives) for studies included in the diagnostic test accuracy meta-analysis. TP = true positive; FP = false positive; FN = false negative; TN = true negative; CVMI = cervical vertebral maturation index; UMGS = University of Michigan Elementary and Secondary School Growth Study Diagnostic positivity thresholds were defined a priori based on circumpubertal CVM stages in each study. Data were extracted directly or reconstructed from reported sensitivity, specificity, and sample size.

Study (Author, Year)	Population / cohort	Study design	Index test (threshold)	Reference standard	Unit of analysis	TP	FP	FN	TN
Perinetti et al., 2016 [9]	UMGS longitudinal cohort; adolescents	Longitudinal	CVMI (CS3-CS4 positive)	Peak annual mandibular growth increment (Co-Gn)	Annual intervals	19	7	3	73
Perinetti et al., 2018 [10]	Oregon & Burlington Growth Studies	Longitudinal	CVMI (CS3-CS4 positive)	Maximum annual Co-Gn increment	Annual intervals	12	30	38	170
Morris et al., 2019 [11]	Orthodontic growth sample	Longitudinal diagnostic study	CVMI (C3 positive)	Mandibular growth peak (P vs NP)	Age-band intervals	14	24	15	114

For studies evaluating the association between SOS maturation and cervical vertebral maturation, individual Pearson correlation coefficients ranged from r = 0.618 to r = 0.890, indicating moderate to strong correlations (Table 3).

**Table 3:** Correlation coefficients between spheno-occipital synchondrosis maturation and cervical vertebral maturation index across included studies. Sex-specific data from Kocasarac et al. [16] were treated as independent subgroups, as reported by the authors. Correlation coefficients were pooled using Fisher’s Z transformation and a random-effects model. CVMI = cervical vertebral maturation index; SOS = spheno-occipital synchondrosis; CBCT = cone-beam computed tomography

Study (Author, Year)	Population / setting	Study design	N	Age (years)	Sex (M/F)	SOS imaging	SOS staging system	CVM/CVMI method	Correlation type	r value	p-value
Fernández-Pérez et al., 2016 [15]	Multicenter orthodontic patients	Cross-sectional	315	6-23	167/148	CBCT	Bassed (5-stage)	Baccetti CVMI	Spearman	0.890	<0.001
Fayad et al., 2020 [17]	Orthodontic clinic sample	Cross-sectional	117	8-18	NR	CBCT	Franklin & Flavel (4-stage)	Baccetti CVMI	Spearman	0.852	<0.001
Kocasarac et al., 2017 [16] (Males)	Turkish sample	Cross-sectional	43	8-28	43/0	CBCT	Franklin & Flavel	Hassel-Farman	Spearman	0.851	<0.001
Kocasarac et al., 2017 [16] (Females)	Turkish sample	Cross-sectional	73	8-28	0/73	CBCT	Franklin & Flavel	Hassel-Farman	Spearman	0.618	<0.001

Synthesis of Results

**Figure 4:**
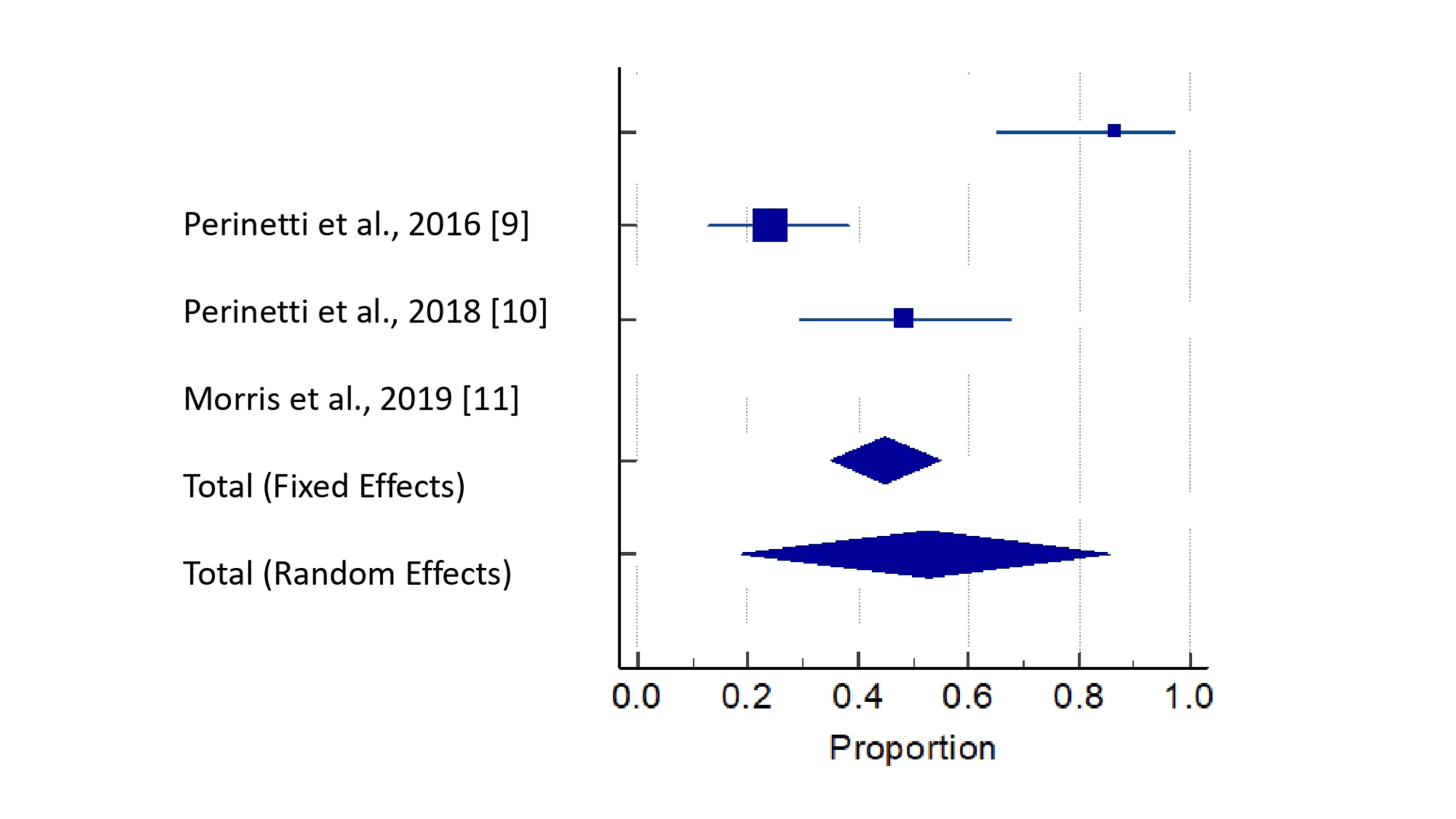
Forest plot showing individual study sensitivity estimates with 95% confidence intervals for cervical vertebral maturation index (CVMI) in identifying the mandibular growth peak, along with pooled estimates using fixed-effect and random-effects models. Substantial heterogeneity was observed among studies, reflected by the wide confidence interval of the random-effects summary estimate.

Diagnostic test accuracy meta-analysis (CVMI): A random-effects meta-analysis demonstrated a pooled sensitivity of 52.8% (95% CI: 18.7%-85.4%) for CVMI in identifying the mandibular growth peak (Figure 4). Considerable heterogeneity was observed (I² = 92.7%, p < 0.001), indicating substantial between-study variability. Fixed-effect and random-effects models yielded notably different pooled estimates, reflecting underlying clinical and methodological heterogeneity among the included studies.

**Figure 5:**
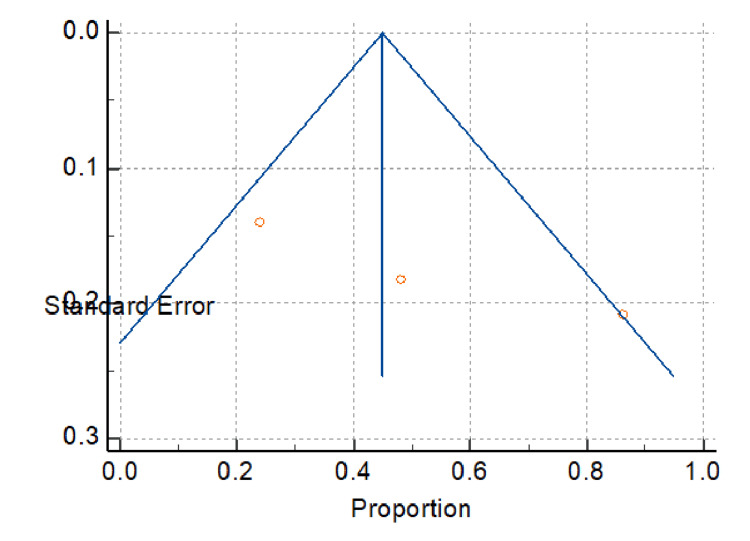
Funnel plot of sensitivity estimates for assessment of publication bias in the diagnostic accuracy meta-analysis. The plot demonstrates no clear evidence of asymmetry, suggesting a low likelihood of small-study effects or publication bias.

Assessment of small-study effects did not demonstrate statistically significant asymmetry based on Egger’s test (p = 0.17) or Begg’s test (p = 0.12) (Figure 5); however, these results should be interpreted cautiously given the small number of included studies.

**Figure 6:**
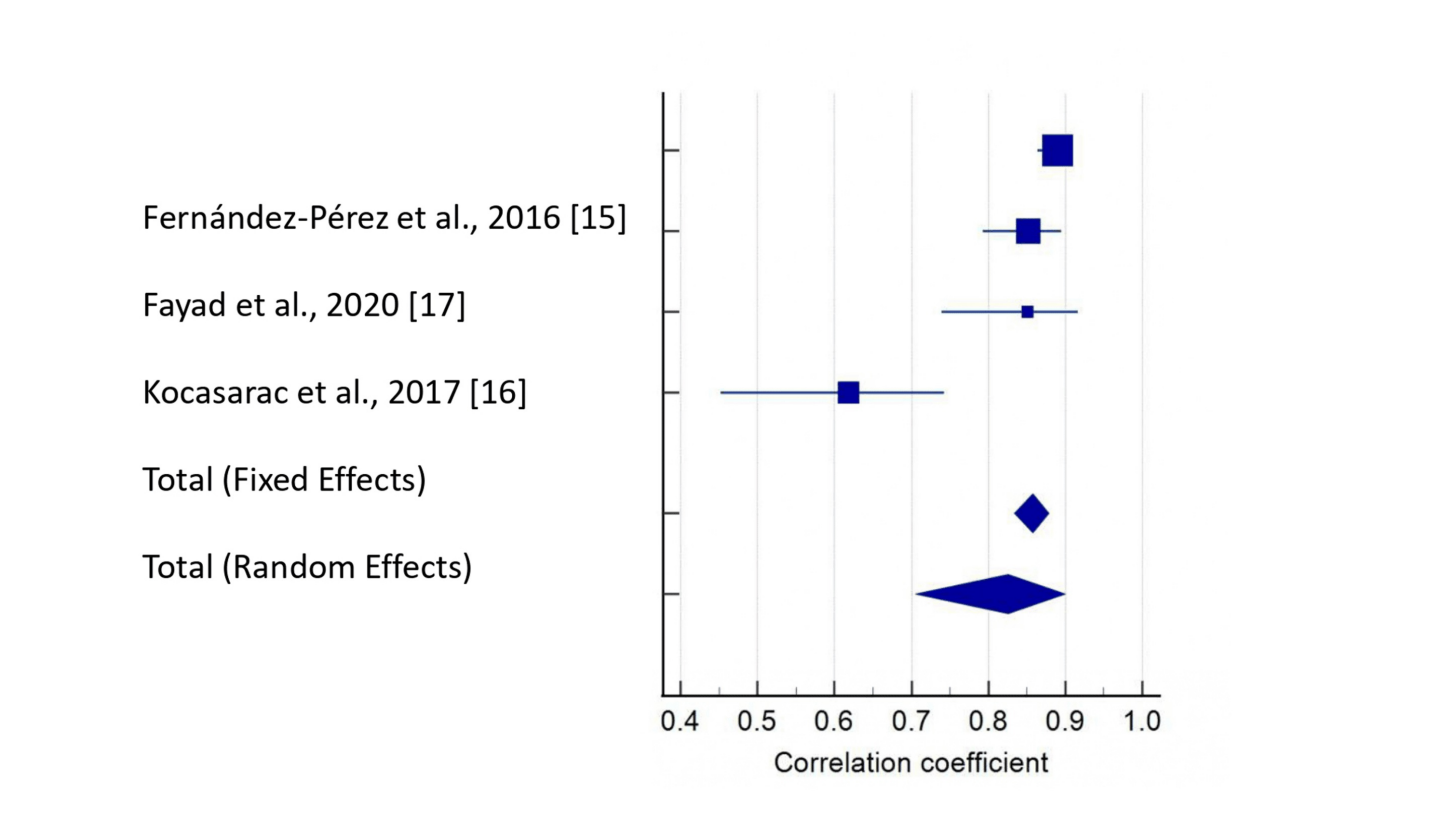
Forest plot of Pearson correlation coefficients demonstrating the association between spheno-occipital synchondrosis maturation and cervical vertebral maturation index stages.

SOS-CVMI correlation meta-analysis: Three studies comprising a total of 548 participants were included in the correlation meta-analysis, with four datasets analyzed because Kocasarac et al. contributed separate male and female subgroup data [15-17]. Under a random-effects model, the pooled Pearson correlation coefficient was r = 0.83 (95% CI: 0.71-0.90; p < 0.001), indicating a strong positive association between SOS maturation and CVMI (Figure 6).

Substantial between-study heterogeneity was observed (I² = 89.3%, p < 0.001), suggesting variability in study populations, imaging protocols, and reference standards. Assessment of publication bias using Egger’s regression test (p = 0.32) and Begg’s rank correlation test (p = 0.50) did not indicate statistically significant asymmetry; however, these results should be interpreted cautiously given the limited number of included studies.

Additional analyses: No subgroup analyses or meta-regression were performed due to the limited number of included studies. Sensitivity analyses excluding individual studies did not materially alter the direction or magnitude of pooled estimates.

No adverse events or inconclusive test results were reported in the included studies.

Discussion

Summary of Evidence

This systematic review and meta-analysis evaluated the diagnostic and associative performance of skeletal maturation indicators used for the assessment of mandibular growth timing. Three longitudinal studies were eligible for DTA synthesis of the CVMI, while three cross-sectional studies contributed to the meta-analysis of the association between SOS maturation and CVMI. In that correlation meta-analysis, four datasets were analyzed because one study contributed separate male and female subgroup data.

The diagnostic accuracy synthesis demonstrated that CVMI exhibits moderate sensitivity for identifying the mandibular growth peak, with a pooled sensitivity of approximately 53%. However, substantial heterogeneity was observed across studies, reflecting differences in study design, growth assessment intervals, and definitions of the growth peak. These findings are consistent with previously reported concerns regarding variability in CVMI performance in longitudinal growth studies [12-14].

In contrast, the SOS-CVMI correlation meta-analysis revealed a strong positive association, with a pooled correlation coefficient exceeding 0.80. This suggests that SOS fusion stages assessed using CBCT are closely aligned with cervical vertebral maturation stages, supporting the biological plausibility of SOS maturation as a marker of skeletal maturity [15-17]. Importantly, applicability concerns were low across studies, indicating that the included populations and imaging modalities were clinically relevant to orthodontic growth assessment.

Overall, the strength of evidence supports SOS maturation as a promising adjunctive indicator of skeletal maturity, while highlighting the limitations of CVMI when used alone for precise identification of the mandibular growth peak.

Limitations

Several limitations should be considered when interpreting these findings. First, the number of longitudinal studies eligible for diagnostic accuracy synthesis was limited, restricting the ability to perform full bivariate HSROC modeling. Consequently, pooled analyses focused on sensitivity estimates rather than combined sensitivity and specificity, which may limit the completeness of diagnostic performance evaluation.

Second, substantial heterogeneity was observed in both diagnostic accuracy and correlation analyses. This heterogeneity likely reflects differences in study populations, growth assessment intervals, imaging protocols, and definitions of reference standards. Although random-effects models were employed to account for between-study variability, residual heterogeneity remains an important consideration.

Third, most SOS studies were cross-sectional in design and relied on CVMI as the reference standard rather than direct longitudinal mandibular growth measurements. While this approach is common in the existing literature, it introduces potential reference standard bias, as reflected by some concerns in the reference standard domain of the QUADAS-2 assessment.

Finally, formal assessment of publication bias did not demonstrate statistically significant asymmetry; however, these results should be interpreted cautiously due to the small number of included studies, which limits the power of statistical tests for detecting small-study effects.

## Conclusions

Within the limitations of the available evidence, CVMI demonstrates moderate diagnostic sensitivity for identifying the mandibular growth peak, with considerable variability across longitudinal studies. In contrast, SOS maturation assessed using CBCT shows a strong and consistent association with CVMI stages, supporting its potential role as an adjunctive skeletal maturity indicator.

These findings suggest that SOS fusion assessment may enhance skeletal maturity evaluation when used alongside established methods, particularly in cases where cervical vertebral assessment is equivocal. However, the current evidence base does not support the use of SOS maturation as a standalone diagnostic test for growth peak identification.

Future research should prioritize well-designed longitudinal studies that directly relate SOS maturation stages to serial mandibular growth outcomes, enabling robust diagnostic accuracy analyses and direct comparison with existing maturation indices. Such evidence will be essential to clarify the intended clinical role of SOS assessment in orthodontic treatment timing and growth modification strategies.
